# A genome-wide cell-free DNA methylation analysis identifies an episignature associated with metastatic luminal B breast cancer

**DOI:** 10.3389/fcell.2022.1016955

**Published:** 2022-10-25

**Authors:** Aitor Rodriguez-Casanova, Nicolas Costa-Fraga, Clara Castro-Carballeira, Miriam González-Conde, Carmen Abuin, Aida Bao-Caamano, Tomás García-Caballero, Elena Brozos-Vazquez, Carmela Rodriguez-López, Victor Cebey, Patricia Palacios, Juan F. Cueva, Rafael López-López, Clotilde Costa, Angel Díaz-Lagares

**Affiliations:** ^1^ Epigenomics Unit, Cancer Epigenomics, Translational Medical Oncology Group (ONCOMET), Health Research Institute of Santiago de Compostela (IDIS), University Clinical Hospital of Santiago (CHUS/SERGAS), Santiago de Compostela, Spain; ^2^ Roche-Chus Joint Unit, Translational Medical Oncology Group (ONCOMET), Health Research Institute of Santiago (IDIS), Santiago de Compostela, Spain; ^3^ Universidade de Santiago de Compostela (USC), Santiago de Compostela, Spain; ^4^ Department of Oncology, Marqués de Valdecilla University Hospital, Santander, Spain; ^5^ Centro de Investigación Biomédica en Red Cáncer (CIBERONC), ISCIII, Madrid, Spain; ^6^ Department of Morphological Sciences, University of Santiago de Compostela and Xerencia de Xestión Integrada de Santiago (XXIS/SERGAS), Santiago de Compostela, Spain; ^7^ Translational Medical Oncology Group (ONCOMET), Health Research Institute of Santiago de Compostela (IDIS), University Clinical Hospital of Santiago (CHUS/SERGAS), Santiago de Compostela, Spain

**Keywords:** DNA methylation, EPIC Array, cell-free DNA, liquid biopsy, metastasis, luminal B, breast cancer

## Abstract

Breast cancers of the luminal B subtype are frequent tumors with high proliferation and poor prognosis. Epigenetic alterations have been found in breast tumors and in biological fluids. We aimed to profile the cell-free DNA (cfDNA) methylome of metastatic luminal B breast cancer (LBBC) patients using an epigenomic approach to discover potential noninvasive biomarkers. Plasma cfDNA was analyzed using the Infinium MethylationEpic array in a cohort of 14 women, including metastatic LBBC patients and nontumor controls. The methylation levels of cfDNA and tissue samples were validated with droplet digital PCR. The methylation and gene expression data of 582 primary luminal breast tumors and 79 nontumor tissues were obtained from The Cancer Genome Atlas (TCGA). We found an episignature of 1,467 differentially methylated CpGs that clearly identified patients with LBBC. Among the genes identified, the promoter hypermethylation of *WNT1* was validated in cfDNA, showing an area under the ROC curve (AUC) of 0.86 for the noninvasive detection of metastatic LBBC. Both paired cfDNA and primary/metastatic breast tumor samples showed hypermethylation of *WNT1*. TCGA analysis revealed significant *WNT1* hypermethylation in the primary tumors of luminal breast cancer patients, with a negative association between *WNT1* methylation and gene expression. In this proof-of-principle study, we discovered an episignature associated with metastatic LBBC using a genome-wide cfDNA methylation approach. We also identified the promoter hypermethylation of *WNT1* in cfDNA as a potential noninvasive biomarker for luminal breast cancer. Our results support the use of EPIC arrays to identify new epigenetic noninvasive biomarkers in breast cancer.

## Introduction

Breast cancer (BC) is the most frequently diagnosed cancer in women worldwide, with 2.3 million new cases (11.7% of all cancer cases) in 2020, representing the leading cause of cancer death in women (Sung et al., 2021). BC is a heterogeneous disease with several distinct clinical characteristics that, according to a gene expression profile, can be divided into four molecular subtypes: luminal, HER2-enriched, basal-like, and normal breast-like (Perou et al., 2000). In addition, luminal tumors can be divided into the luminal A and B subtypes according to the expression profile of the estrogen receptor (ER), progesterone (PR), HER2, and proliferation tumor status (Cheang et al., 2009). The luminal B subtype is a common BC subtype characterized by high proliferation, resistance to standard therapies, risk of early relapse, and poor prognosis (Tran and Bedard, 2011; Cornen et al., 2014; Li et al., 2016). In addition, this tumor subtype is more likely to exhibit local recurrence and single bone metastases than nonluminal BC. However, recent studies have not investigated this tumor subtype as thoroughly as other subtypes (Li et al., 2016). Notably, the incidence of luminal B tumors has increased in recent years in many racial/ethnic and age groups (Acheampong et al., 2020).

Cancer metastasis is characterized by highly variable clinical manifestations and is responsible for over 90% of cancer-related deaths (Gupta and Massague, 2006; Chaffer and Weinberg, 2011). However, despite recent advances, the clinical need to identify biomarkers in metastatic BC disease remains unmet (Gupta et al., 2020). In recent years, liquid biopsy has emerged as a good opportunity to address this clinical need. This noninvasive approach allows for the characterization of the molecular landscape of circulating tumor elements in body fluids, such as epigenetic modifications of cell-free DNA (cfDNA), to obtain biomarkers for the management of cancer patients (Siravegna et al., 2017).

The most well-known epigenetic modification is DNA methylation, which is an important regulator of gene expression originating from the addition of a methyl group (CH_3_) to the 5’ carbon of cytosines in cytosine–phosphate–guanine (CpG) dinucleotides (Bao-Caamano et al., 2020). The deregulation of this epigenetic mechanism in breast tumor cells has major implications for cancer development, progression, and therapy response (Gomez-Miragaya et al., 2019; Martin-Pardillos et al., 2019; Palomeras et al., 2019; Pineda et al., 2019). Notably, the analysis of DNA methylation in liquid biopsy has shown utility as a potential clinical biomarker for BC patients (Palanca-Ballester et al., 2021).

Recently published studies of other tumor types have shown that epigenomic approaches based on the Infinium MethylationEPIC array (EPIC array) technology, which covers over 850,000 CpG sites along the human genome, could be useful to profile the methylation of cfDNA in biological fluids (Gallardo-Gomez et al., 2018; Herrgott et al., 2022). Therefore, this proof-of-principle study aimed to profile the cfDNA methylome of luminal B breast cancer (LBBC) patients using an EPIC array approach to discover new noninvasive biomarkers. In this study, we identified an epigenetic signature (episignature) based on the methylation of cfDNA associated with metastatic LBBC. Among the genes of this episignature, we confirmed the hypermethylation of *WNT1* in cfDNA and tumor tissues (primary and metastatic) as a potential new biomarker for LBBC patients. The results of our work support the application of the EPIC array technology as a noninvasive tool to identify new biomarkers in breast cancer.

## Methods

### Study participants

In this retrospective study, 9 luminal B metastatic breast cancer patients and 5 healthy controls (nontumor controls) were recruited between 2016 and 2018 at the Medical Oncology Department at the University Clinical Hospital of Santiago de Compostela (Spain). Most of the metastatic patients of this study (7 out of 9) had been diagnosed in the past at M0 stage. Two patients of our cohort had metastases at the time of primary tumor diagnosis. The study was approved by the Galician Ethical Committee (reference number 2015/772) and conducted in accordance with the guidelines for Good Clinical Practice and the Declaration of Helsinki. All participants included in the study signed the informed consent to participate.

### Blood and tissue samples

Blood sample was obtained from all the patients at the time of diagnosis of metastasis and before starting the treatment. Blood samples were collected by phlebotomy into collection tubes containing K_2_EDTA as an anticoagulant. Plasma was isolated within 2 h of collection by initial centrifugation at 1,700 × g for 10 min at room temperature (RT), followed by a second centrifugation at 15,000 × g for 10 min at RT. Isolated plasma was stored at −80°C until analysis. All tumor tissues used were obtained according to standard-of-care (SOC) procedures. We used formalin-fixed and paraffin-embedded (FFPE) primary and/or metastatic tumor and matched nontumor tissue samples available from 4 patients included in the study. Whole slide FFPE tissue sections of 10 μm were obtained.

### Isolation of DNA from plasma and tissue samples

We used the QIAamp^®^ Circulating Nucleic Acid Kit (Qiagen) and the vacuum system QIAvac 24 Plus (Qiagen) following the manufacturer´s recommendations to isolate cfDNA from 2 ml of plasma. DNA was also isolated from 10-μm FFPE tissue sections using the AllPrep DNA/RNA FFPE Kit (Qiagen) following the manufacturer’s protocol. The quality and quantity of DNA from FFPE tissue sections were evaluated with a NanoDrop (Thermo Fisher), and cfDNA was quantified using the Qubit 1× dsDNA High-Sensitivity Assay Kit and a Qubit 4.0 Fluorometer (Thermo Fisher Scientific). The DNA from FFPE tissue sections and cfDNA from plasma were stored at −80°C until analysis.

### Genome-wide cell-free DNA methylation analysis

Fifteen nanograms of each individual sample of plasma cfDNA was bisulfite-converted using the EZ DNA Methylation Lightning Kit (Zymo Research) following the manufacturer’s recommendations. Subsequently, the bisulfite-modified cfDNA was then subjected to whole genome amplification (WGA) using the EpiTect Whole Bisulfitome Kit (Qiagen) according to the manufacturer’s protocol. Briefly, the bisulfite-modified cfDNA of each individual sample was amplified with a reaction buffer containing REPLI-g Midi DNA Polymerase (Qiagen) at 28°C for 8 h, which was subsequently inactivated at 95 °C for 5 min. After the WGA of cfDNA, the Illumina Infinium HD methylation protocol was followed using MethylationEPIC BeadChips that were analyzed in a HiScan (Illumina). Samples with a mean detection *p*-value < 0.01 were considered valid for the analysis. The methylation data were processed in the R statistical environment using RnBeads 2.0 (Muller et al., 2019). Raw intensity data files (IDATs) were imported into RnBeads 2.0 for quality control and preprocessing. First, a greedycut algorithm was used to filter out low-quality probes. Probes overlapping with SNPs and probes whose sequences mapped to multiple genomic locations (cross-reactive) were removed. IDATs obtained in the array were normalized using the beta-mixture quantile (BMIQ) method. Hierarchical linear models were used to obtain the methylation differences between groups. *p*-values were corrected for multiple testing using the Benjamini‒Hochberg method, and a false discovery rate (FDR) < 10% was selected for significance. The DNA methylation level was represented as the average β-value, which was calculated as the ratio of the fluorescent signal intensity of the methylated probe to those of total (methylated and unmethylated) probes. Average β-values were used to calculate the mean methylation difference between groups as the Δβ-value (β-value Luminal B – β-value Control). An unsupervised hierarchical clustering heatmap of β-values was generated using the ComplexHeatmap package. Gene ontology (GO) enrichment analysis of biological pathways from the PANTHER database was performed using GENECODIS (Tabas-Madrid et al., 2012).

### Methylation and expression analysis from The Cancer Genome Atlas

The DNA methylation (β-values) and expression data of *WNT1* in luminal primary breast tumors and nontumor controls were obtained from the public datasets of The Cancer Genome Atlas (TCGA) Cancer Genome Atlas, N. (2012). The breast cancer subtype of patients was obtained from the clinical information available at TCGA and the classification of these TCGA patients based on PAM50 assay performed by Netanely et al. (2016).

### Methylation analysis of the *WNT1* promoter in cell-free DNA by droplet digital PCR

The methylation of the *WNT1* promoter was analyzed by droplet digital PCR (ddPCR) in a QX200 system (Bio-Rad). Twenty nanograms of plasma cfDNA and 30 ng of DNA from FFPE tissue samples were bisulfite converted using the EZ DNA Methylation Lightning Kit (Zymo Research) following the manufacturer’s recommendations. A custom Bio-Rad assay to detect the methylation status of *WNT1* (cg27196808) was designed: *WNT1*-M for methylation and *WNT1*-U for unmethylation (Supplementary Table S1). A multiplex preamplification reaction was performed with ∼2 ng of bisulfite-converted DNA using SsoAdvanced™ PreAmp Supermix (Bio-Rad), *WNT1*-M, and *WNT1*-U. The PCR conditions were as follows: 3 min at 95°C, 10 cycles of 95°C for 15 s and 50.6°C for 4 min, and a final hold step at 4°C. Next, a multiplex reaction mix was prepared with 2 µL of the preamplification product using ddPCR Supermix for Probes (No dUTP) (Bio-Rad), *WNT1*-M, and *WNT1*-U. The QX200™ Droplet Generator (Bio-Rad) was used to generate droplets. The thermocycling conditions were as follows: 10 min at 95°C, 40 cycles of 95°C for 15 s and 50.6°C for 30 s, 98°C for 10 min, and a final hold step at 4°C. The temperature ramp increment was 2.5°C/s for all steps. Droplets were counted and analyzed using the QX200™ Droplet Reader system (Bio-Rad), and the QuantaSoft analysis software (Bio-Rad) was used to acquire data. Water was included as a no-template control, and the Human Methylated and Non-Methylated DNA set (Zymo Research) was used as a positive control for methylation and unmethylation. Reactions were performed in triplicate. DNA methylation was expressed according to the following formula: Methylation (%) = [M/(U + M)] x 100, where M represents the copies/μl of methylated cfDNA, and U the copies/μl of unmethylated cfDNA.

### Statistical analysis

The Kolmogorov‒Smirnov test was used to evaluate the normality of the distribution of the data. The nonparametric Mann‒Whitney U test was used to compare methylation data. To assess the diagnostic accuracy, a receiver operating characteristic (ROC) curve was generated. The greatest combination of sensitivity and specificity was obtained using the Youden index (J): J = sensitivity + specificity - 1. The association between DNA methylation and gene expression was evaluated with a Spearman correlation. The GraphPad Prism 6.0 software (GraphPad Software) and the R statistical environment (version 4.2.0) were used for statistical analysis and graphical representation. All expressed *p*-values were calculated with two-tailed tests and were considered significant when the *p*-value < 0.05.

## Results

### Clinical characteristics of patients

A retrospective cohort of 14 women was included in this study: 9 patients with LBBC at the time of metastatic disease diagnosis and 5 nontumor controls. The mean age of the patients was 66 ± 16 years, whereas the control group had a mean age of 53 ± 10 years. The main clinical characteristics of the analyzed cohort are described in Supplementary Table S2. All patients had distant metastases and invasive ductal carcinoma with a high Ki-67 proliferative index (≥20%), and they were positive for estrogen receptors (ER+). Eight out of nine patients were positive for progesterone receptors (PR+), and two patients had HER2 overexpression (Supplementary Table S2). Six out of the 9 patients (66%) included in the study had lung metastasis, 4 patients (44%) showed bone lesions, and 3 patients (33%) had liver affectation. In addition, 5 of the patients (55%) had multiple metastatic locations.

### Genome-wide cell-free DNA methylation analysis of metastatic patients with luminal B breast cancer

**FIGURE 1:**
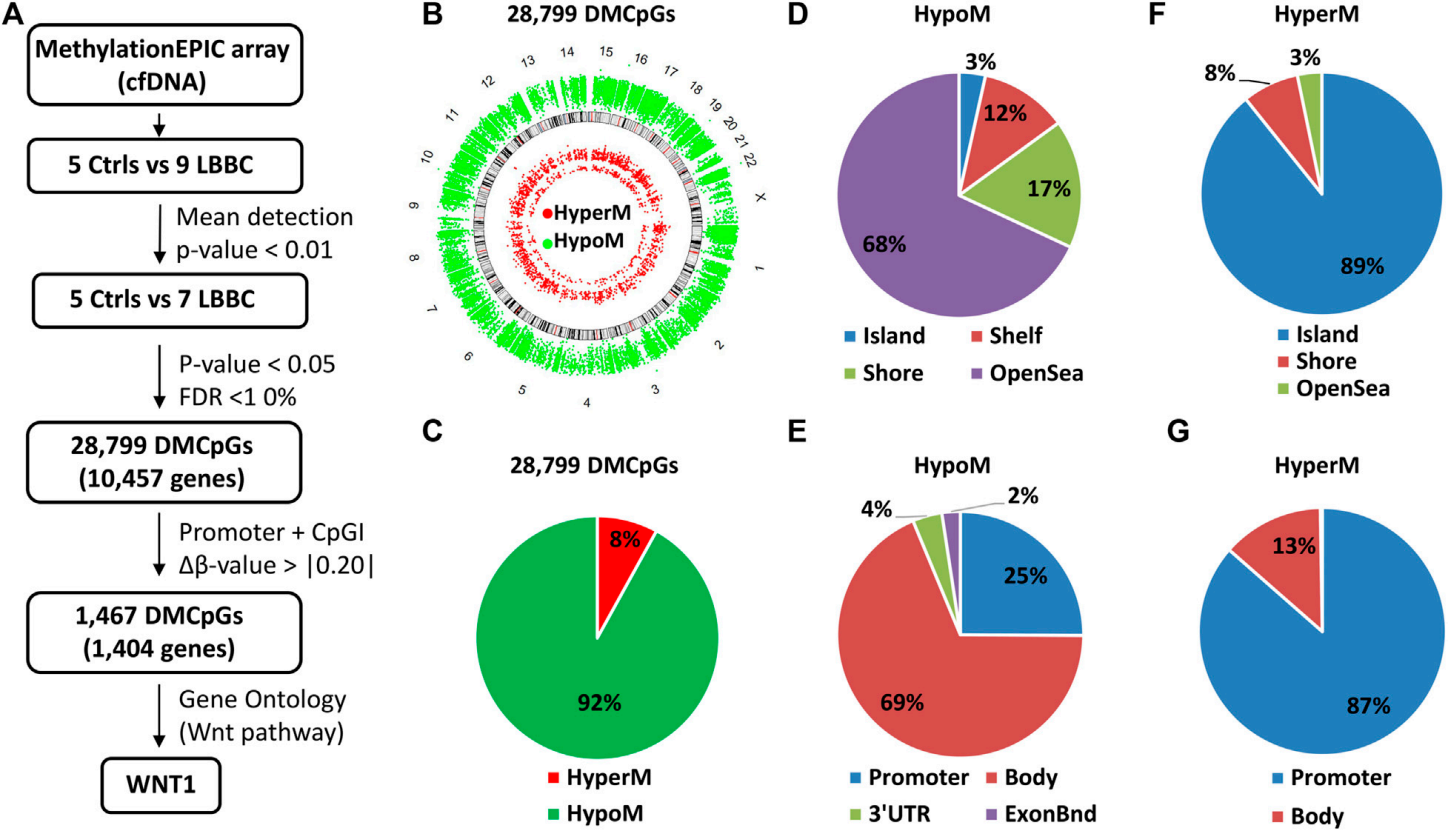
Methylation landscape of cell-free DNA in metastatic patients with luminal B breast cancer. **(A)** Flowchart of the cfDNA methylation analysis. Individual cfDNA samples from controls and luminal B breast cancer patients were analyzed with EPIC array. **(B–G)** Description of the 28,799 differentially methylated CpGs (DMCpGs) found in cfDNA of luminal B breast cancer patients according to **(B)** chromosome location, **(C)** methylation status, **(D,F)** CpG context, and **(E,G)** gene location. cfDNA, cell-free DNA; Ctrls, controls; LBBC, luminal B breast cancer; FDR, false discovery rate; HypoM, hypomethylated; HyperM, hypermethylated.

The analysis of DNA methylome with the EPIC array methodology usually needs a high amount of DNA, which is difficult to obtain in the clinic from individual plasma samples. As a novelty in our study, to overcome this limitation, we have used small amounts of cfDNA from individual plasma samples, which were genome-wide amplified after bisulfite modification and then analyzed using EPIC arrays. Thus, using this approach we performed a genome-wide cell-free DNA methylation analysis in our cohort of 9 LBBC patients and 5 nontumor controls (Figure 1A). After hybridizing the samples in the EPIC array, 2 LBBC samples showed a mean detection *p*-value > 0.01 and were not considered valid for the analysis. Therefore, we ultimately compared the methylation status of cfDNA in 7 LBBC patients and 5 nontumor controls, leading to 28,799 differentially methylated CpGs (DMCpGs) (*p* < 0.05; FDR < 10%) between LBBC and nontumor controls. These DMCpGs showed a wide distribution throughout all chromosomes of the genome (Figure 1B). Of these DMCpGs, 92% (26,486) were hypomethylated and 8% (2,313) were hypermethylated in LBBC patients with respect to nontumor controls (Figure 1C). Most of the hypomethylated CpGs were distributed in regions with low CpG density (open sea) (Figure 1D) and outside promoter regions (Figure 1E), whereas hypermethylated CpGs were mainly located in CpG islands (CpGIs) (Figure 1F) and promoters (Figure 1G).

### Identification of a cell-free DNA episignature in metastatic patients with luminal B breast cancer

**FIGURE 2:**
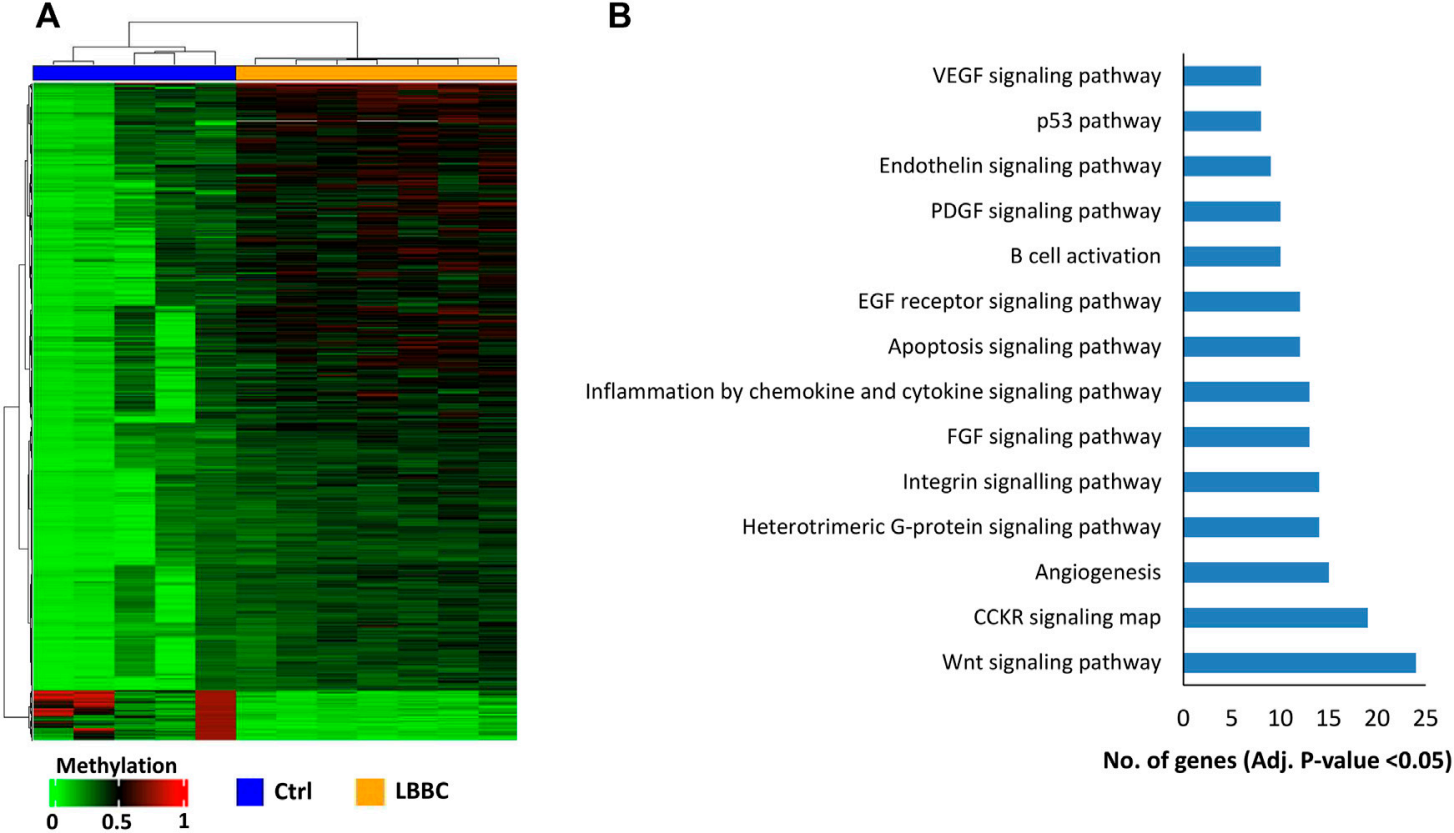
Episignature of cell-free DNA in metastatic patients with luminal B breast cancer. **(A)** Unsupervised hierarchical clustering heatmap of the episignature (1,467 DMCpGs) obtained in cfDNA that differentiates LBBC patients (*n* = 7) from nontumor controls (*n* = 5). **(B)** Gene Ontology enrichment analysis by the PANTHER database, showing the most representative pathways associated with the episignature of cfDNA in luminal B breast cancer patients. Ctrls, controls; LBBC, luminal B breast cancer; DMCpGs, differentially methylated CpGs; cfDNA, cell-free DNA.

The aberrant hypermethylation of CpGI promoters is a very relevant feature that usually occurs in tumor cells (Baylin and Chen, 2005). Therefore, we focused our study on analyzing the methylation profile of cfDNA at the CpGIs of promoters. In these regions of cfDNA, we identified 1,467 DMCpGs (*p* < 0.05; FDR < 10%) with a difference in methylation (Δβ-value) higher than 0.20 (Δβ-value > |0.20|) (Figure 1A). Notably, this epigenetic signature (episignature) of 1,467 DMCpGs was able to clearly differentiate LBBC patients from nontumor controls (Figure 2A). Next, to obtain information related to the functional pathways involved in the identified episignature, we performed a gene ontology (GO) enrichment analysis based on the PANTHER database. This analysis revealed that methylation differences in the cfDNA of LBBC patients and nontumor controls were mainly associated with genes related to the Wnt signaling pathway (Figure 2B). Table 1 shows the 34 DMCpGs (corresponding to 24 genes) of the episignature of cfDNA that are associated with the Wnt signaling pathway. Relevantly, the genes of these 34 DMCpGs that are associated with Wnt signaling belonged to a network significantly enriched in protein interactions (*p* < 0.001) according to a STRING analysis (Supplementary Figure S1).

**TABLE 1:** The 34 CpGs of cfDNA episignature found in metastatic patients with luminal B breast cancer associated with the Wnt signaling pathway.

TargetID^a^	Chr^b^	Position	Gene name	Gene region	Δβ^c^	*p*-value
cg26821418	9	2016890	*SMARCA2*	5′UTR; 5′UTR; 5′UTR	0.35	0.0028
cg27201625	10	6622279	*PRKCQ*	TSS200	0.33	0.0008
cg04351665	10	6622297	*PRKCQ*	TSS200	0.21	0.0057
cg03306374	16	23847325	*PRKCB*	1stExon; 5′UTR; 5′UTR	0.28	0.0024
				1stExon		
cg06931245	8	28351501	*FZD3*	TSS1500	0.26	0.0023
cg18463655	8	28351544	*FZD3*	TSS200; TSS200	0.23	0.0041
cg26631144	8	30670260	*PPP2CB*	5′UTR; 1stExon; 5′UTR	0.28	0.0014
				1stExon		
cg02478409	6	33589019	*ITPR3*	TSS200	0.32	0.0020
cg16490096	1	40367661	*MYCL1*	1stExon; 5′UTR; 5′UTR	0.23	0.0024
				1stExon; 5′UTR; 1stExon		
cg20462899	1	40367831	*MYCL1*	TSS200; TSS200; TSS200	0.29	0.0056
**cg02771661**	**12**	**49372162**	***WNT1***	**TSS200**	**0.21**	**0.0047**
**cg27196808**	**12**	**49372281**	***WNT1***	**1stExon;5′UTR**	**0.22**	**0.0066**
cg13469346	3	53195186	*PRKCD*	TSS200; TSS200	0.24	0.0026
cg21950287	19	54385441	*PRKCG*	TSS200	0.23	0.0051
cg13885159	11	62473858	*GNG3*	TSS1500	0.23	0.0064
cg03922588	11	62473871	*GNG3*	TSS1500	0.27	0.0021
cg25220961	17	64298782	*PRKCA*	TSS200	0.27	0.0023
cg11676500	17	64298789	*PRKCA*	TSS200	0.23	0.0026
cg08221093	16	68119222	*NFATC3*	TSS200; TSS200; TSS200	0.25	0.0067
				TSS200		
cg21367137	16	68119381	*NFATC3*	5′UTR; 5′UTR; 5′UTR	0.26	0.0046
				1stExon; 1stExon; 1stExon		
cg22722737	9	82187628	*TLE4*	5′UTR; 5′UTR; 5′UTR; 5′UT; 1stExon; 1stExon; 1stExon; 1stExon; 1stExo; 5′UTR	0.22	0.0050
cg26753733	4	102268824	*PPP3CA*	TSS200; TSS200; TSS200	0.24	0.0039
cg08764167	10	103113933	*BTRC*	5′UTR; 5′UTR; 1stExon	0.21	0.0050
				1stExon		
cg20359285	2	119603969	*EN1*	1stExon	0.21	0.0043
cg00557469	5	133562427	*PPP2CA*	TSS1500	0.25	0.0049
cg18671773	5	141016477	*HDAC3*	TSS200	0.27	0.0060
cg16248329	4	187644739	*FAT1*	5′UTR	0.23	0.0026
cg02968914	19	1955395	*CSNK1G2*	5′UTR	−0.32	0.0042
cg01895482	19	2556145	*GNG7*	5′UTR	−0.35	0.0015
cg07223632	22	46930499	*CELSR1*	1stExon	−0.36	0.0028
cg00875636	22	46931138	*CELSR1*	1stExon	−0.28	0.0030
cg27334938	18	77167042	*NFATC1*	5′UTR	−0.30	0.0051
cg02113385	18	77203443	*NFATC1*	5′UTR	−0.35	0.0060
cg27475132	4	187645120	*FAT1*	TSS200	−0.23	0.0053

^a^ CpGs located in CGIs of promoters (TSS1500, TSS200, 5′UTR, 1st exon).

^b^ Chromosome.

^c^ Δβ-values (β-value Luminal B - β-value Control). CpGs of the gene *WNT1* are indicated in bold.

### Hypermethylation of the *WNT1* promoter in the cell-free DNA and tumor samples of patients with luminal B breast cancer

**FIGURE 3:**
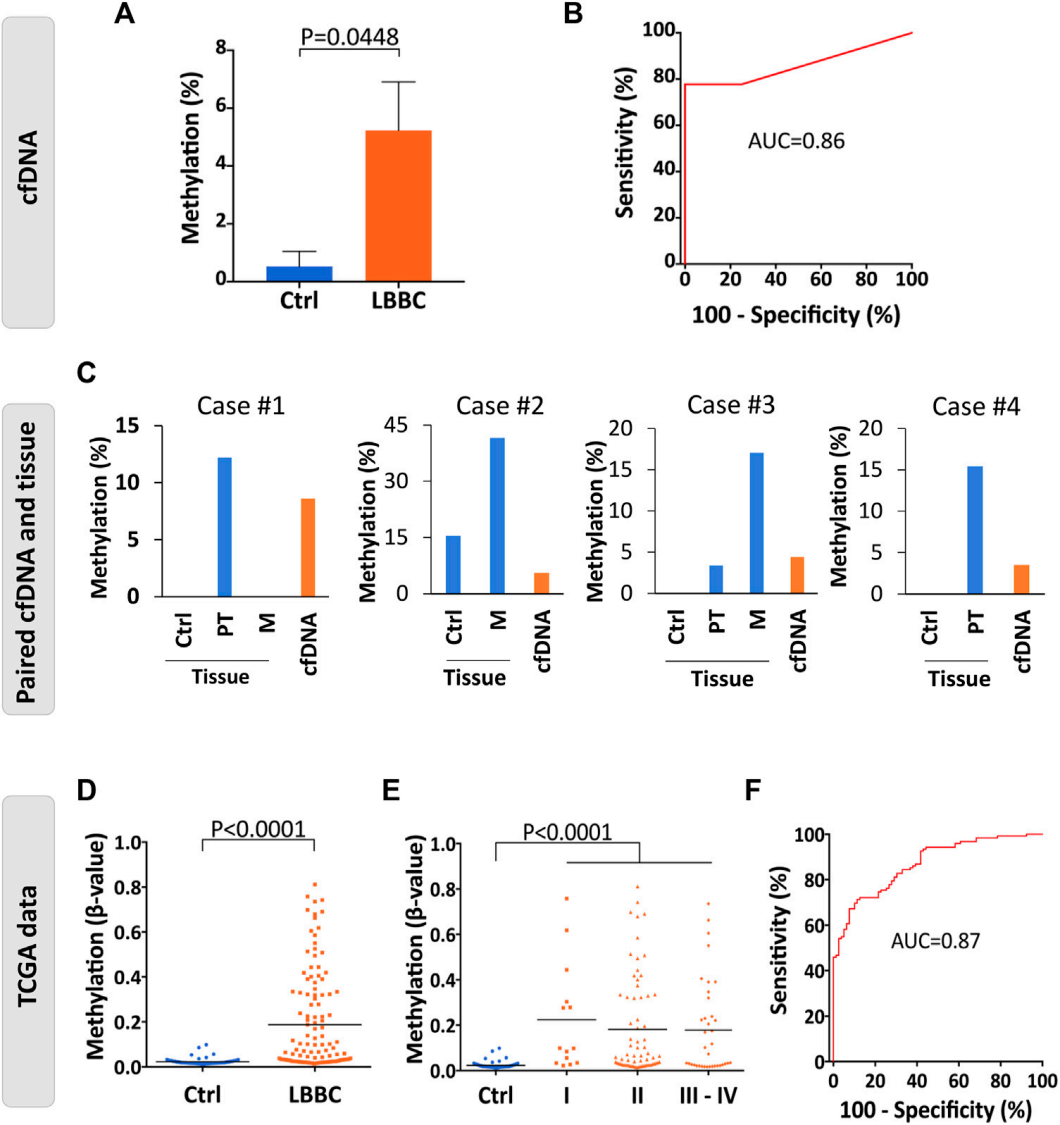
Methylation status of the *WNT1* promoter in the cfDNA and tumor samples of patients with luminal B breast cancer. **(A)** Validation of the methylation levels of the *WNT1* promoter (cg27196808) in cell-free DNA (cfDNA) of luminal B breast cancer patients (*n* = 9) and nontumor controls (*n* = 4) analyzed by droplet digital PCR (ddPCR). Methylation levels are represented as the mean ± SEM. **(B)** Diagnostic accuracy of the methylation of the *WNT1* promoter using droplet digital PCR (ddPCR) in cfDNA for the detection of metastatic luminal B breast cancer patients (*n* = 9) with respect to nontumor controls (*n* = 4). **(C)** Methylation levels of *WNT1* in cfDNA and paired breast primary and/or metastatic tumor samples of 4 luminal B breast cancer patients analyzed by droplet digital PCR (ddPCR). Nontumor tissues from the same patients were used as controls. **(D–E)** Methylation status of *WNT1* in primary tumors of luminal B breast cancer patients (*n* = 122) and nontumor controls (*n* = 79) analyzed by 450K methylation array and obtained from TCGA considering **(D)** all TNM stages together (stages I–IV, *n* = 122) or **(E)** separated according to TNM stage (I, *n* = 14; II, *n* = 70; III-IV, *n* = 37). The horizontal line represents the mean methylation values. **(F)** ROC curve evaluating the methylation of *WNT1* to detect primary tumors of luminal B breast cancer patients (stages I-IV, *n* = 122) with respect to nontumor controls (*n* = 79) from TCGA. Ctrl, control; P, *p*-value; AUC, area under the ROC curve; ROC curve, receiver operating characteristic curve. PT, primary tumor; M, metastasis.

Among the DMCpGs in the episignature obtained from the cfDNA of LBBC patients that were associated with the Wnt signaling pathway (Table 1), we found 2 CpGs (cg27196808 and cg02771661) located in *WNT1* that were hypermethylated in the cfDNA of LBBC patients with respect to nontumor controls. To confirm this aberrant methylation, we selected the most DMCpG of *WNT1* (cg27196808), and we analyzed its methylation status in the cfDNA of our cohort using ddPCR. As expected, the methylation of *WNT1* was significantly higher in LBBC patients than in nontumor controls (Figure 3A). In addition, using a ROC curve analysis, the methylation status of the *WNT1* promoter analyzed by ddPCR accurately differentiated LBBC patients from nontumor controls, with an area under the ROC curve (AUC) of 0.86 (95% CI: 0.65–1.00, *p* = 0.045) (Figure 3B), a sensitivity of 78% (CI 95%: 40%–98%), and a specificity of 100% (CI 95%: 40%–100%).

To verify that the hypermethylation of *WNT1* found in cfDNA (cg27196808) was also present in the tumor tissues of patients with LBBC, we assessed its methylation status by ddPCR in the available matched primary and/or metastatic tumor tissue samples (*n* = 4) of our cohort. This assay revealed that *WNT1* hypermethylation was present not only in the cfDNA but also in the paired primary and/or metastatic tumor samples of the LBBC patients analyzed (Figure 3C). Next, we took advantage of public DNA methylation array data from The Cancer Genome Atlas (TCGA) to evaluate whether the hypermethylation of the *WNT1* promoter was a frequent event in LBBC. This analysis showed that the methylation of the *WNT1* promoter (cg27196808) was significantly higher in luminal B primary tumors (stages I–IV) than in nontumor controls (Figure 3D), and this observation was consistent across all TNM tumor stages (Figure 3E). However, the methylation status of *WNT1* did not differ among the tumor stages of LBBC analyzed (Figure 3E). An ROC curve analysis showed that *WNT1* methylation differentiated luminal B primary tumors (stages I-IV) from nontumor controls with high diagnostic accuracy, with an AUC of 0.87 (95% CI: 0.82–0.92, *p* < 0.0001) (Figure 3F). In addition, we also analyzed the *WNT1* expression data (RNAseq) available from LBBC patients and nontumor controls included in TCGA, revealing that *WNT1* was significantly downregulated in this BC subtype (Supplementary Figure S2).

Finally, we also evaluated in breast primary tumors from TCGA whether the hypermethylation of *WNT1* was a specific event of LBBC patients. The methylation levels of *WNT1* were significantly higher in LBBC than in the other breast tumor subtypes (Supplementary Figure S3). Interestingly, we observed that *WNT1* was also significantly hypermethylated in other breast cancer subtypes (Luminal A, triple negative and HER2+) in comparison with nontumor controls.

## Discussion

Alterations in epigenetic mechanisms, such as aberrant DNA methylation, are implicated in the development, progression, and therapy response of BC (Palomeras et al., 2019; Pineda et al., 2019; Glodzik et al., 2020). In recent years, the methylation analysis of liquid biopsy samples in BC patients has shown clinical utility as a biomarker for the detection, prognosis, and monitoring of the disease (Hoque et al., 2006; Mastoraki et al., 2018; Palanca-Ballester et al., 2021). However, a clinical need to find new noninvasive biomarkers associated with metastatic BC subtypes persists (Gupta et al., 2020). Herein, we focused our study on patients with advanced LBBC, since it represents a frequent, aggressive and poor prognosis BC subtype (Creighton, 2012). The characterization of liquid biopsy samples using epigenomic tools for genome-wide methylation analyses has been recently proposed as a good approach to discover new noninvasive biomarkers (Shen et al., 2018). Thus, we used a genome-wide DNA methylation approach based on the EPIC array methodology to profile the methylome of cfDNA and discover novel noninvasive biomarkers in metastatic LBBC patients.

Our work revealed that the cfDNA of patients with metastatic LBBC is characterized by the hypomethylation of regions with a low density of CpGs and the site-specific hypermethylation of CpGIs in promoter regions. Importantly, this pattern is similar to the deregulation of DNA methylation that has been previously described in cancer cells (Nishiyama and Nakanishi, 2021), suggesting that the methylation profile in the cfDNA of the patients in our cohort mirrors the epigenetic alterations of BC cells.

Specific genes, such as *RASSF1A* and *BRCA1*, have been previously described to exhibit aberrant hypermethylation of their promoter CpGIs in BC (Rice et al., 1998; Dammann et al., 2001). Accordingly, we focused our study on promoter CpGIs and were able to identify a novel noninvasive episignature in cfDNA based on 1,467 CpGs that was associated with LBBC patients. We found that the genes of this episignature were related to relevant biological pathways, mainly Wnt signalling pathway. Among these genes, we focused on *WNT1*, which is involved in the canonical Wnt signaling pathway (also known as Wnt/β-Catenin) in cancer cells (Ayyanan et al., 2006; Mehta et al., 2021). Thus, we confirmed that the promoter CpGI of *WNT1* was hypermethylated in the cfDNA of patients with metastatic LBBC and that this aberrant methylation showed a high diagnostic accuracy to detect this BC subtype, suggesting that the hypermethylation of *WNT1* could be a suitable biomarker for cancer detection and monitoring of metastatic patients. In line with this, it has been recently shown that methylation biomarkers of cfDNA with high diagnostic accuracy are useful not only for diagnosis but also for monitoring tumor burden dynamics under different therapeutic regimens in advanced disease (Barault et al., 2018). Importantly, evaluating prognosis and monitoring tumor response in real time during treatment continues to be an unmet clinical need in advanced BC (Gupta et al., 2020). Wnt signaling is a very relevant pathway in BC whose molecular alterations have clinical implications to establish the prognosis of the disease (Mukherjee et al., 2012) and has been associated to breast cancer therapy response (Abreu de Oliveira et al., 2022). Therefore, it would be interesting to evaluate in future studies whether the hypermethylation of *WNT1* could be useful for the selection of patients susceptible to systemic therapies (CDK inhibitors for example) in the BC metastatic setting.

Of note, we also found that the promoter hypermethylation of *WNT1* was present not only in cfDNA of LBBC patients but also in their primary and/or metastatic tumors. This finding is in accordance with our previous work and that of other authors showing that the molecular landscape present in liquid biopsy may also be detected in the corresponding tumor tissue of patients (Rahvar et al., 2020; Rodriguez-Casanova et al., 2021). In addition, when we extended our study to the analysis of breast primary tumors using the public TCGA database, we were able to confirm that the hypermethylation of *WNT1* is a frequent event in early and advanced LBBC, suggesting that the epigenetic deregulation of *WNT1* is not a specific biomarker of metastatic disease but rather a biomarker of breast cancer cells. In agreement with our results, the aberrant methylation of other genes involved in the Wnt signaling pathway (e.g., *WNT5A* and *WNT7A*) has previously been described in tumor cells from the BC luminal subtype (Shan et al., 2019) and in other tumor types, such as gastric cancer or chronic lymphocytic leukemia (Liu et al., 2019; Poppova et al., 2022).

Several studies have shown that aberrant promoter hypermethylation is a relevant mechanism that is able to repress the expression of key genes in breast tumor cells (Alvarez et al., 2013; Palomeras et al., 2019). Accordingly, the analysis of luminal breast tumors from the TCGA database also revealed that *WNT1* promoter hypermethylation was associated with a downregulation of its gene expression in primary tumors, suggesting that *WNT1* is epigenetically regulated in luminal BC. The downregulation of *WNT1* observed in particular BC subtypes corroborates the work by Koval and Katanaev (2018), who reported low expression of this gene in primary tumors of nontriple-negative BC patients (ER+/PR + and HER+). Indeed, it has been reported that the deregulation of some Wnt signaling components depends on the BC subtype, with many being downregulated in the luminal B subtype (Smid et al., 2008).

One limitation of our study is that the epigenomic profiling of cfDNA performed is based on a retrospective cohort of patients with a small sample size. Although the results obtained in this work should be taken with caution, they provide the basis for further large, prospective and independent studies that validate the clinical utility of the potential epigenetic biomarkers identified herein. In addition, it would be interesting to evaluate in future works the implications of the epigenetic deregulation of *WNT1* in the development of metastasis.

In summary, in this proof-of-principle study, we discovered an episignature associated with patients with advanced LBBC using a genome-wide cfDNA methylation approach. We also identified the promoter hypermethylation of *WNT1* in cfDNA as a potential noninvasive biomarker for luminal BC. Our results support the use of EPIC array technology to identify new noninvasive biomarkers in BC.

## Data Availability

The methylation data of cfDNA analyzed in this study with EPIC Array are available at the NCBI GEO repository with accession number GSE214344.
