# Supplementary material for: A genome-wide cell-free DNA methylation analysis identifies an episignature associated with metastatic luminal B breast cancer
Source: Front Cell Dev Biol. 2022 Oct 25;10:1016955. doi: 10.3389/fcell.2022.1016955 (PMC9641197; doi:10.3389/fcell.2022.1016955)
Supplement: Supplementary file 1 [file DataSheet1.pdf]

Supplementary Material

1 Supplementary Figures and Tables

1.1 Supplementary Tables

Supplementary Table 1. List of primers used for the methylation analysis of *WNT1* by ddPCR.

**Assay**	**Primers/Probe**	**5´-Sequence-3´**
WNT1-M	Forward	GAGGGGTAGTTTTTTTT
	Reverse	TCTAACTTTAACAACCCTAAA
	Probe (FAM)	GATCGCGAGTTATGTTGT
WNT1-U	Forward	GAGGGGTAGTTTTTTTT
	Reverse	TCTAACTTTAACAACCCTAAA
	Probe (HEX)	GGATTGTGAGTTATGTTGTT

Supplementary Table 2. Clinical characteristics of metastatic patients with luminal B breast cancer included in the study.

Characteristics	Patients (n=9)	
	No.	%
**Age (years)**		
<65	3	33
65-75	3	33
>75	3	33
**Immunohistochemistry**		
PR+	8	89
ER+	9	100
HER2+	2	22
Ki-67 ≥ 20%	9	100
**Histology**		
Ductal	9	100
**Stage**		
IV	9	100
**Metastatic sites**		
1	4	44
2	2	22
3	3	33
**Metastatic location**		
Visceral	6	66
Bone	4	44

1.2 Supplementary Figures

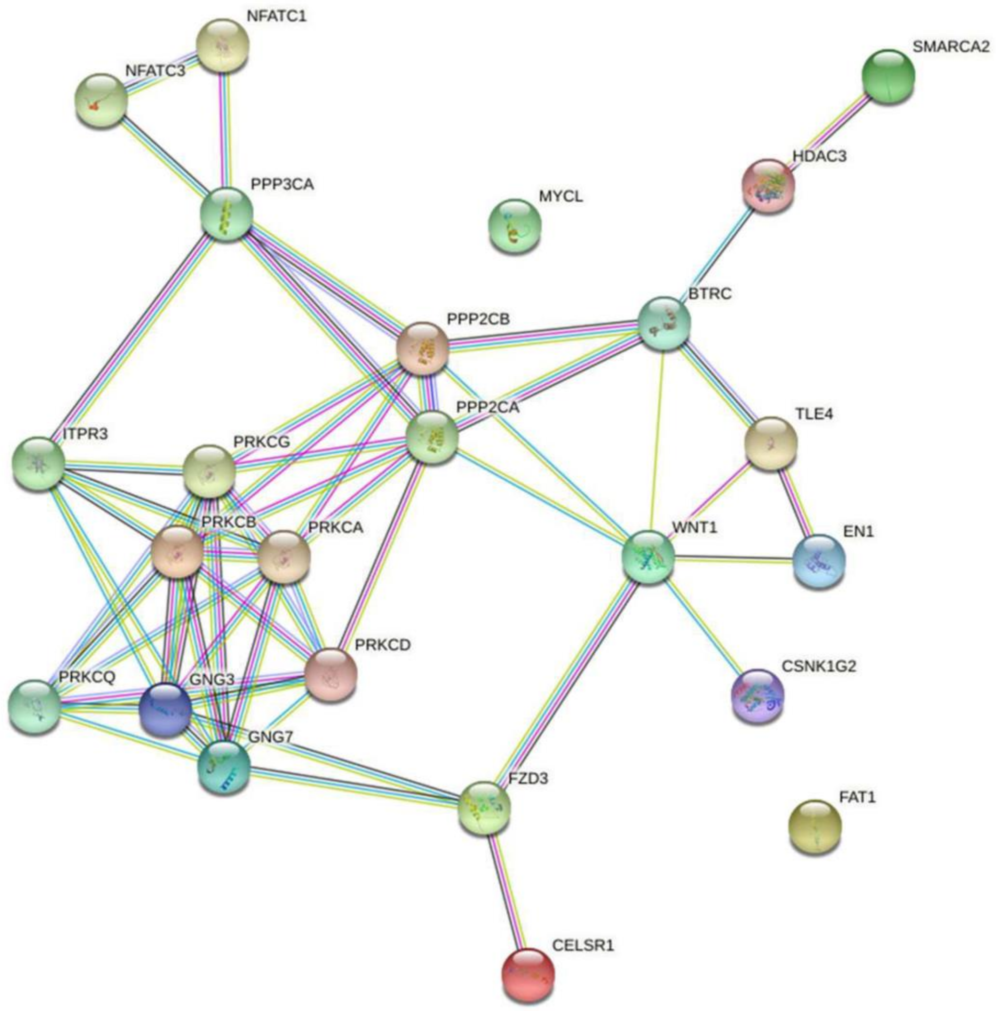

Supplementary Figure 1. The genes of the 34 DMCpGs related to the Wnt signaling pathway belong to a functional network significantly enriched in protein interactions (P<0.001).

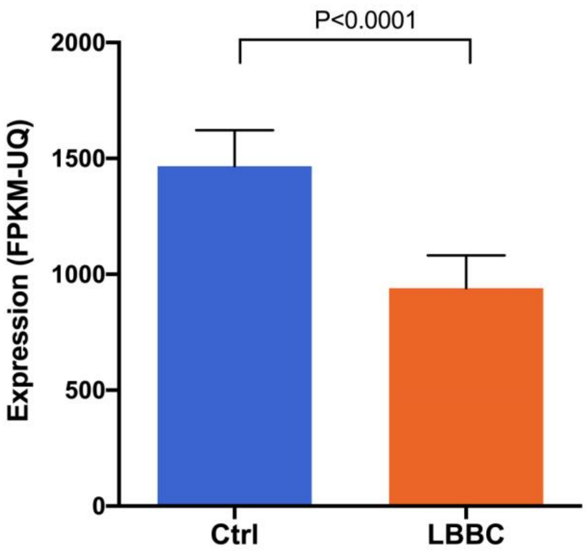

Supplementary Figure 2. Expression of *WNT1* in primary luminal B breast tumors. Gene expression levels of *WNT1* available in primary tumors of luminal B breast cancer patients (n=122) and nontumor controls (n=67) obtained by RNA-seq from TCGA. Expression levels are represented as the mean ± SEM.

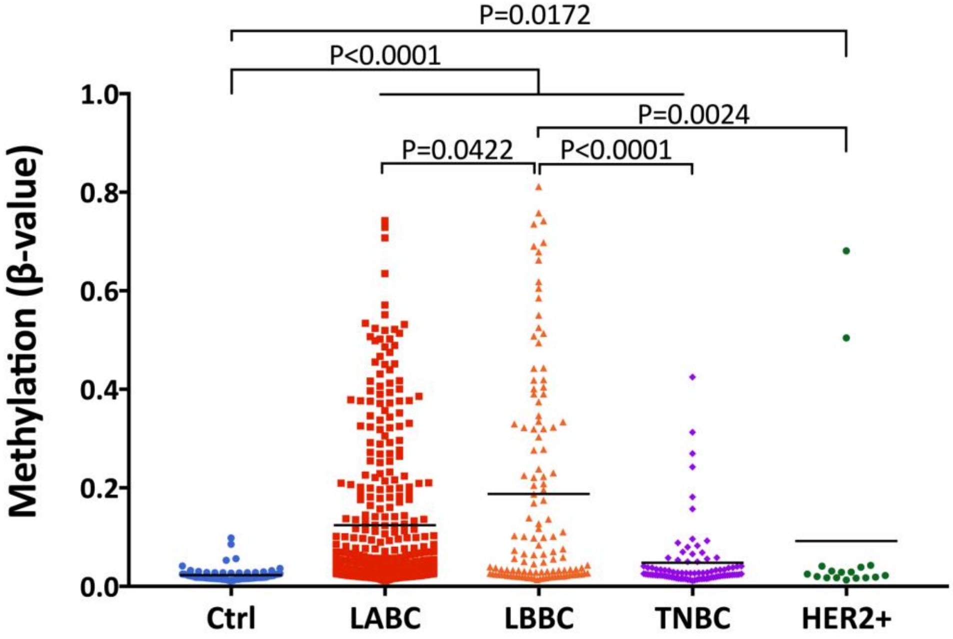

Supplementary Figure 3. Methylation status of *WNT1* in primary tumors of breast cancer subtypes (LABC, n=358; LBBC, n=122; TNBC, n=87; HER2+, n=17) and nontumor controls (n=79) from TCGA. Methylation data have been obtained from EPIC array analysis considering all TNM stages together (stages I-IV). Ctrl, control; P, p-value. LABC, luminal A breast cancer; LBBC, Luminal B breast cancer; TNBC, triple negative breast cancer; HER2+, HER2-positive.
